# An artificial intelligence-designed predictive calculator of conversion from minimally invasive to open colectomy in colon cancer

**DOI:** 10.1007/s13304-024-01915-2

**Published:** 2024-06-26

**Authors:** Sameh Hany Emile, Nir Horesh, Zoe Garoufalia, Rachel Gefen, Peter Rogers, Steven D. Wexner

**Affiliations:** 1 Ellen Leifer Shulman and Steven Shulman Digestive Disease Center, Cleveland Clinic Florida, 2950 Cleveland Clinic Blvd., Weston, FL 33179 USA; 2 Colorectal Surgery Unit, General Surgery Department, Mansoura University Hospitals, Mansoura, Egypt; 3 Department of Surgery and Transplantation, Sheba Medical Center, Ramat-Gan, Israel; 4 Department of General Surgery, Faculty of Medicine, Hadassah Medical Organization, Hebrew University of Jerusalem, Jerusalem, Israel

**Keywords:** Predictors, Impact, Conversion, Minimally Invasive colectomy, Open colectomy, Colon cancer

## Abstract

Minimally invasive surgery is safe and effective in colorectal cancer. Conversion to open surgery may be associated with adverse effects on treatment outcomes. This study aimed to assess risk factors of conversion from minimally invasive to open colectomy for colon cancer and impact of conversion on short-term and survival outcomes. This case–control study included colon cancer patients undergoing minimally invasive colectomy from the National Cancer Database (2015–2019). Logistic regression analyses were conducted to determine independent predictors of conversion from laparoscopic and robotic colectomy to open surgery. 26,546 patients (mean age: 66.9 ± 13.1 years) were included. Laparoscopic and robotic colectomies were performed in 79.1% and 20.9% of patients, respectively, with a 10.6% conversion rate. Independent predictors of conversion were male sex (OR: 1.19, *p* = 0.014), left-sided cancer (OR: 1.35, *p* < 0.001), tumor size (OR: 1, p = 0.047), stage II (OR: 1.25, *p* = 0.007) and stage III (OR: 1.47, *p* < 0.001) disease, undifferentiated carcinomas (OR: 1.93, *p* = 0.002), subtotal (OR: 1.25, *p* = 0.011) and total (OR: 2.06, p < 0.001) colectomy, resection of contiguous organs (OR: 1.9, *p* < 0.001), and robotic colectomy (OR: 0.501, *p* < 0.001). Conversion was associated with higher 30- and 90-day mortality and unplanned readmission, longer hospital stay, and shorter overall survival (59.8 vs 65.3 months, *p* < 0.001). Male patients, patients with bulky, high-grade, advanced-stage, and left-sided colon cancers, and patients undergoing extended resections are at increased risk of conversion from minimally invasive to open colectomy. The robotic platform was associated with reduced odds of conversion. However, surgeons’ technical skills and criteria for conversion could not be assessed.

**Supplementary Information:**

The online version contains supplementary material available at 10.1007/s13304-024-01915-2.

## Introduction

Minimally invasive surgery (MIS) has gained increasing popularity in various surgical disciplines, including colorectal surgery [1, 2]. The increased uptake of MIS is attributable to its documented benefits which include smaller abdominal incisions that are associated with less tissue trauma, less pain, and lower rates of wound-related complications. In addition, MIS has been associated with expedited recovery and shorter hospital stays [3].

Conversion from minimally invasive colorectal surgery to an open approach is undesired, yet remains a potential event. Conversion to open surgery can be reactive or preemptive. A prior study from our department  [4] found that reactive conversion is associated with higher complications and longer hospital stays than preemptive conversion, recommending having a low threshold for preemptive conversion rather than the need for reactive conversion. A meta-analysis [5] reported the pooled rate of conversion from laparoscopic to open surgery in colorectal cancer to be approximately 18%. The authors found male sex, rectal cancers, and advanced tumor stage to be associated with a higher risk of conversion to open surgery. A national database analysis [6] reported a lower conversion rate of 15.8% and found laparoscopic transverse colectomy to have the highest conversion rate of 20.8% followed by left colectomy at 20.7%. The rates of conversion from minimally invasive to open colorectal surgery may vary according to the type and approach of resection. It has been noted that minimally invasive proctectomy has a higher conversion rate compared to minimally invasive colectomy. Another study reported the highest conversion rate (31.2%) with laparoscopic proctectomy, whereas right colectomy had the lowest rate (12.9%) [7]. Recent reports documented lower conversion rates with robotic-assisted proctectomy [8] and colectomy [9] than their laparoscopic counterparts.

The impact of conversion from MIS to open surgery has been investigated and it has been shown that unplanned conversion was associated with increased rates of short-term morbidity and mortality, longer hospital stays, and worse disease-free survival (DFS) [10]. Although conversion is associated with a higher mortality rate than patients who had their MIS successfully completed (1.4% vs 0.6%), converted patients still had lower mortality rates than patients who had a planned open surgery (1.4% vs 3.9%) [7].

The majority of previous studies [11] investigated the risk factors of conversion of minimally invasive colectomy and proctectomy together, but few studies [12–14] have specified the risk factors for conversion in colectomy alone. These studies included small numbers of patients who were converted to open surgery. Since proctectomy and colectomy each entail different technical aspects and possibly different risk factors for conversion to open surgery, we opted to assess the risk factors of conversion from minimally invasive to open colectomy for colon cancer and the impact of conversion on short-term and survival outcomes. The hypothesis of the study is that some risk factors for conversion might be modifiable, thus knowing these factors beforehand may lower the incidence of conversion to open surgery.

## Patients and methods

### Study design and setting

This study was a case–control analysis of patients with stage I-III colonic adenocarcinoma who were treated with minimally invasive, either laparoscopic or robotic-assisted, colectomy. The National Cancer Database (NCDB) was accessed between 2015 and 2019 to obtain the data used in this study. The NCDB includes data from more than 1500 Commission on Cancer (CoC)-accredited hospitals across the United States and is a joint project of the CoC of the American College of Surgeons and the American Cancer Society. Ethics committee approval and written consent to participate in the study were not required since the study was a retrospective review of de-identified patient data from a public database.

Cases were defined as patients who were converted from minimally invasive to open colectomy and controls were patients who had their minimally invasive colectomy successfully completed. Conversion was defined using the parameter “Approach-Surgery of the Primary Site at this Facility” with codes 2 and 4 used to indicate when the surgery began as robotic-assisted or laparoscopic procedure and was converted to open, whereas codes 1 and 3 indicated the completion of robotic-assisted or laparoscopic surgery without conversion. A baseline comparison of cases and controls was done to assess for factors significantly associated with conversion to unplanned open surgery.

### Study population

The study included patients with stage I–III colonic adenocarcinoma who underwent laparoscopic or robotic colectomy. We excluded the following patients:

o Patients with appendiceal cancers.
o Patients with other pathologic types of colonic cancer.
o Patients with stage 0 or stage IV disease, or those with unknown clinical stage.
o Patients who did not undergo surgery, underwent local excision, or had unknown/non-specified surgery.
o Patients who underwent open surgery or with an unknown surgical approach.

### Data collected

Data used in the present study included:

- Demographics: age, sex, race, Charlson comorbidity index score, insurance status, residence area, and facility type.
- Tumor characteristics: TNM stage, location, size, histologic type, and grade
- Treatments: systemic therapy, type of colectomy, and surgical approach. Types of surgery included segmental colectomy, hemicolectomy/subtotal colectomy, total colectomy, proctocolectomy, and non-specified colectomy.
- Outcomes: conversion to open surgery, hospital stay, 30-day readmission, and 30- and 90-day mortality

### Outcomes

The primary outcome of the study was the rate and predictors of conversion from minimally invasive to open colectomy. The secondary outcomes were the impact of conversion on hospital stay, short-term mortality, 30-day readmission, and overall survival (OS).

### Statistical analysis

Statistical analyses were performed using EZR (version 1.55) and R software (version 4.1.2). Continuous data were expressed in the form of mean and standard deviation when normally distributed or as the median and interquartile range (IQR) and were analyzed with the student *t*-test or Mann–Whitney-*U*. Categorical data were expressed as numbers and absolute proportions and were analyzed using the Fisher exact test or Chi-Square test. A complete case analysis was used to address missing data. The significant factors in the univariate analysis with *p* values less than 0.05 were selected to enter into a binary logistic regression multivariable analysis to determine the independent predictors of conversion. The odds ratio (OR) of the independent predictors of conversion were used to create a predictive calculator for conversion using an R code written by the artificial intelligence (AI)-driven natural language processing tool, ChatGPT. The R code is provided in the Supplementary Material.

The area under the curve (AUC) of the model used was estimated to evaluate the discriminatory ability of the model. Multicollinearity between the predictors included in the model was examined using the variance inflation factor (VIF) where a VIF > 10 implies high multicollinearity. P values < 0.05 were considered significant. “The de-identified data used in the study are derived from the NCDB and its participating hospitals that are not responsible for the statistical validity of the analysis or the conclusions of the study”.

## Results

### Description of the study cohort

**Fig. 1:**
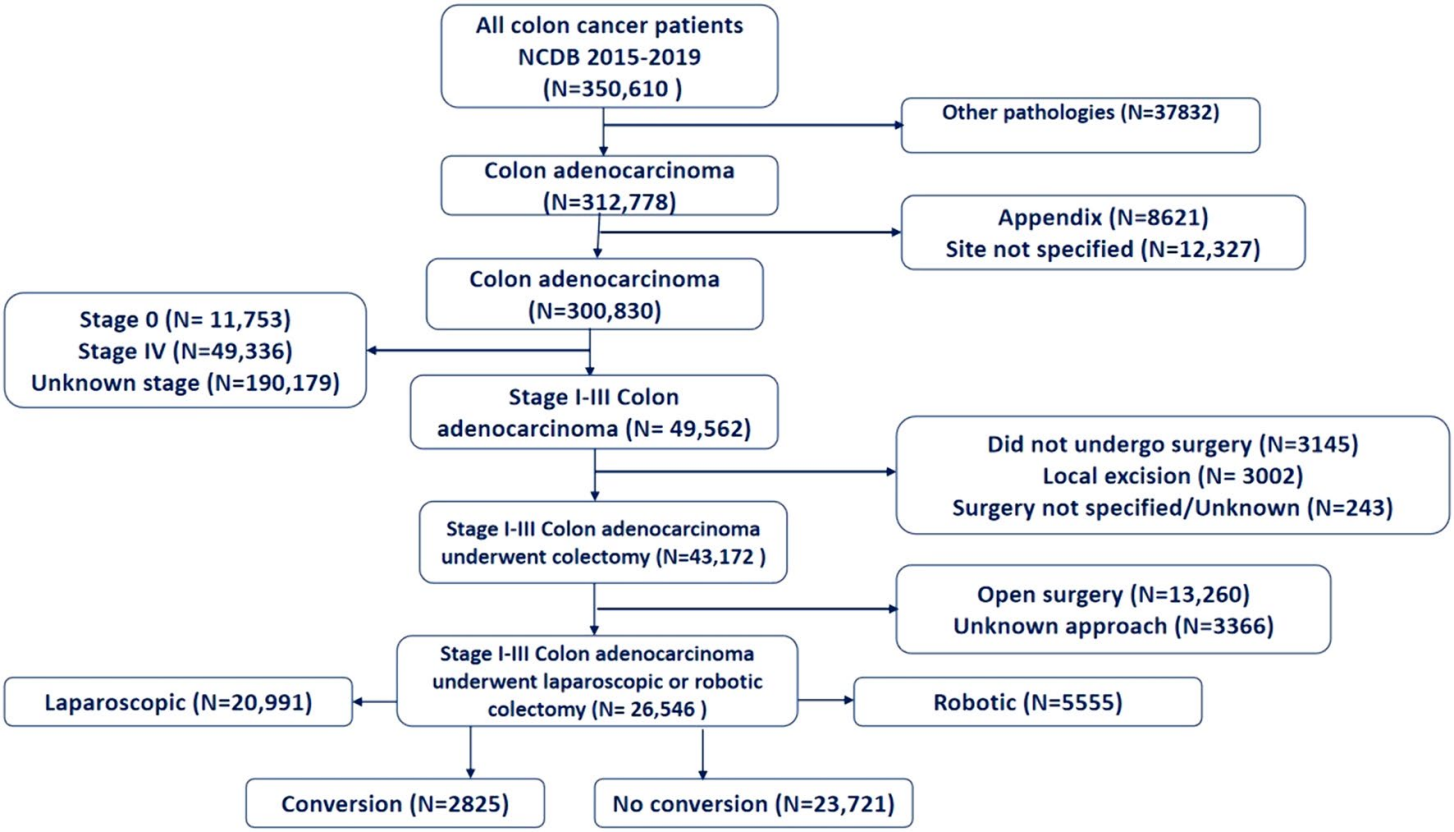
Flow chart illustrating patient selection

After screening the records of 312,778 patients with colonic adenocarcinoma treated between 2015 and 2019, 26,546 patients were included in the study (Fig. 1). The mean age of patients was 66.9 ± 13.1 years, and the male-to-female ratio was 1:1. The majority of patients were White (82.7%), followed by Black (11.6%), and Asian (3.9%). 29.4% of patients had a Charlson score ⋝ 1. Approximately half (49.2%) of the tumors were located on the right side of the colon, 39.5% were located on the left side, and 9.9% in the transverse colon; 1.3% of tumors were overlapping lesions. Most patients were insured by Medicare (54.3%) or private insurance (37.2%) and lived in metropolitan (87.7%) followed by urban (10.9%) areas.

The distribution of clinical TNM stages within the cohort was as follows: Stage I (57.7%), Stage II (25.8%), and Stage III (16.6%). Non-mucinous adenocarcinomas accounted for most colon cancers (91.7%) followed by mucinous adenocarcinomas (7.4%) and signet-ring cell carcinomas (0.9%). The majority of (71.6%) patients did not receive systemic therapy, while 1.1% received neoadjuvant systemic therapy, 26.8% received adjuvant systemic therapy, and 0.5% received both neoadjuvant and adjuvant systemic therapy. The common types of colectomies performed were subtotal colectomy/hemicolectomy (58.0%) followed by segmental colectomy (38.3%), whereas total colectomy and proctocolectomy accounted for 2.3% and 0.5% of cases, respectively. Laparoscopic colectomy was performed in 79.1% of patients and robotic colectomy in 20.9% (Table 1).

**Table 1:** Characteristics of the entire cohort

Factor	Group	Overall
Number		26,546
Mean age in years (SD)		66.91 (13.06)
Sex (%)	Male	13,274 (50.0)
	Female	13,272 (50.0)
Race (%)	White	21,758 (82.7)
	Black	3041 (11.6)
	Asian	1029 (3.9)
	American Indian	96 (0.4)
	Other	397 (1.5)
Hispanic origin (%)	Hispanic	1760 (6.8)
	Non-Hispanic	24,116 (93.2)
Charlson Deyo Score (%)	0	18,729 (70.6)
	1	4841 (18.2)
	2	1604 (6.0)
	3	1372 (5.2)
Tumor location (%)	Right	13,053 (49.2)
	Transverse colon	2615 (9.9)
	Left	10,523 (39.6)
	Overlapping lesion	355 (1.3)
Insurance (%)	Medicaid	1489 (5.7)
	Medicare	14,224 (54.3)
	Other government	227 (0.9)
	Private insurance	9756 (37.2)
	Not insured	509 (1.9)
Residence area (%)	Metropolitan	22,793 (87.7)
	Urban	2830 (10.9)
	Rural	373 (1.4)
Facility type (%)	Academic/Research Program	8050 (31.1)
	Community Cancer Program	1806 (7.0)
	Comprehensive Community Cancer Program	10,856 (41.9)
	Integrated Network Cancer Program	5187 (20.0)
Clinical TNM stage (%)	I	15,308 (57.7)
	II	6843 (25.8)
	III	4395 (16.6)
Histology (%)	Adenocarcinoma	24,344 (91.7)
	Mucinous adenocarcinoma	1956 (7.4)
	Signet-ring cell carcinoma	246 (0.9)
Grade (%)	Well-differentiated	3057 (12.5)
	Moderately differentiated	17,494 (71.4)
	Poorly differentiated	3479 (14.2)
	Undifferentiated	469 (1.9)
Systemic treatment (%)	No systemic therapy	18,874 (71.6)
	Neoadjuvant	292 (1.1)
	Adjuvant	7059 (26.8)
	Neoadjuvant and adjuvant	140 (0.5)
	Intraoperative	4 (0.0)
Type of colectomy (%)	Partial colectomy, segmental resection	10,177 (38.3)
	Subtotal colectomy/hemicolectomy	15,386 (58.0)
	Total colectomy	604 (2.3)
	Total proctocolectomy	137 (0.5)
	Colectomy, non-specified	242 (0.9)
Approach (%)	Laparoscopic	20,991 (79.1)
	Robotic-assisted	5555 (20.9)

*SD* standard deviation

### Factors associated with conversion

Overall, 2825 patients required conversion to open surgery with a rate of 10.6% (95%CI: 10.3–11%). The factors that were significantly associated with conversion were male sex (*p* < 0.001), Charlson score > 1 (*p* = 0.014), race (*p* = 0.006), insurance (*p* < 0.001), residence (*p* < 0.001), facility type (*p* < 0.001), tumor location (p = 0.001), tumor size (*p* < 0.001), clinical TNM stage (p < 0.001), tumor histology (*p* = 0.003) and grade (*p* < 0.001), type of colectomy (*p* < 0.001), resection of a contiguous organ (p < 0.001), and surgical approach (*p* < 0.001) (Table 2).

**Table 2:** Univariate analysis of the factors associated with conversion to open surgery

Factor	Group	No conversion (*n* = 23,721)	Conversion (*n* = 2825)	*p*-value
Mean age in years (SD)		66.91 (13.04)	66.89 (13.30)	0.933
Sex (%)	Male	11,768 (49.6)	1506 (53.3)	** < 0.001**
	Female	11,953 (50.4)	1319 (46.7)	
Charlson Deyo Score (%)	0	16,798 (70.8)	1931 (68.4)	**0.014**
	1	4311 (18.2)	530 (18.8)	
	2	1408 (5.9)	196 (6.9)	
	3	1204 (5.1)	168 (5.9)	
Race (%)	White	19,497 (82.9)	2261 (80.7)	**0.006**
	Black	2658 (11.3)	383 (13.7)	
	Asian	925 (3.9)	104 (3.7)	
	American Indian	84 (0.4)	12 (0.4)	
	Other	354 (1.5)	43 (1.5)	
Hispanic origin (%)	Hispanic	1575 (6.8)	185 (6.7)	0.904
	Non-Hispanic	21,550 (93.2)	2566 (93.3)	
Insurance (%)	Medicaid	1264 (5.4)	225 (8.1)	** < 0.001**
	Medicare	12,713 (54.3)	1511 (54.1)	
	Other government	198 (0.8)	29 (1.0)	
	Private insurance	8800 (37.6)	956 (34.3)	
	Not insured	439 (1.9)	70 (2.5)	
Residence area (%)	Metro	20,415 (87.9)	2378 (85.6)	**0.001**
	Urban	2482 (10.7)	348 (12.5)	
	Rural	320 (1.4)	53 (1.9)	
Facility type (%)	Academic/Research Program	7165 (30.9)	885 (32.2)	** < 0.001**
	Community Cancer Program	1568 (6.8)	238 (8.7)	
	Comprehensive Community Cancer Program	9707 (41.9)	1149 (41.8)	
	Integrated Network Cancer Program	4711 (20.3)	476 (17.3)	
Tumor location (%)	Right	11,761 (49.6)	1292 (45.7)	**0.001**
	Left	9376 (39.5)	1147 (40.6)	
	Transverse colon	2280 (9.6)	335 (11.9)	
	Overlapping lesion	304 (1.3)	51 (1.8)	
Median tumor size in mm (IQR)		40.00 [25.00, 57.00]	45.00 [30.00, 65.00]	** < 0.001**
Clinical TNM stage (%)	I	13,962 (58.9)	1346 (47.6)	** < 0.001**
	II	5949 (25.1)	894 (31.6)	
	III	3810 (16.1)	585 (20.7)	
Histology (%)	Adenocarcinoma	21,794 (91.9)	2550 (90.3)	**0.003**
	Mucinous adenocarcinoma	1720 (7.3)	236 (8.4)	
	Signet-ring cell carcinoma	207 (0.9)	39 (1.4)	
Grade (%)	Well-differentiated	2761 (12.6)	296 (11.1)	** < 0.001**
	Moderately differentiated	15,674 (71.8)	1820 (68.5)	
	Poorly differentiated	3021 (13.8)	458 (17.2)	
	Undifferentiated	386 (1.8)	83 (3.1)	
Neoadjuvant systemic treatment (%)	Yes	383 (1.6)	49 (1.7)	0.636
	No	23,183 (98.4)	2754 (98.3)	
Resection of a contiguous organ (%)	Yes	1193 (5.0)	349 (12.4)	** < 0.001**
	No	22,528 (95.0)	2476 (87.6)	
Type of colectomy (%)	Segmental colectomy	9224 (38.9)	953 (33.7)	** < 0.001**
	Subtotal /hemicolectomy	13,654 (57.6)	1732 (61.3)	
	Total colectomy	517 (2.2)	87 (3.1)	
	Total proctocolectomy	120 (0.5)	17 (0.6)	
	Colectomy, non-specified	206 (0.9)	36 (1.3)	
Approach (%)	Laparoscopic	18,498 (78.0)	2493 (88.2)	** < 0.001**
	Robotic	5223 (22.0)	332 (11.8)	

*IQR* interquartile range

Bold text in *p* value column indicates statistical significance

### Predictors of conversion

The independent predictors of conversion were male sex (OR: 1.19, *p* = 0.014), left-sided cancer (OR: 1.35, *p* < 0.001), tumor size (OR: 1, *p* = 0.047), stage II disease (OR: 1.25, *p* = 0.007), stage III disease (OR: 1.47, *p* < 0.001), undifferentiated carcinomas (OR: 1.93, *p* = 0.002), subtotal colectomy (OR: 1.25, *p* = 0.011) or total colectomy (OR: 2.06, *p* < 0.001), resection of contiguous organs (OR: 1.9, *p* < 0.001), and robotic colectomy (OR: 0.501, *p* < 0.001) (Table 3). The area under the curve of the model used was 0.627 (95%CI: 0.609- 0646). No evidence of multicollinearity between the predictors of conversion was found as the VIF of the factors examined ranged between 1 and 1.05.

**Table 3:** Multivariate analysis of the predictors of conversion to open surgery

Variable	Group	Odds ratio	Lower 95%CI	Upper 95%CI	*p*-value
Sex	Female	1	–	–	–
	Male	1.19	1.04	1.37	**0.014**
Race	White	1	–	–	–
	Black	1.05	0.852	1.3	0.633
	Asian	0.764	0.518	1.13	0.176
	American Indian	0.739	0.171	3.2	0.686
	Other	0.946	0.576	1.55	0.826
Charlson Deyo score	0	1	–	–	–
	1	1.17	0.992	1.39	0.062
	2	1.31	0.999	1.72	0.051
	3	1.21	0.864	1.68	0.272
Residence area	Metropolitan	1	–	–	–
	Urban	1.19	0.953	1.49	0.124
	Rural	1.21	0.708	2.05	0.491
Insurance	Medicaid	1	–	–	–
	Medicare	0.72	0.547	0.946	**0.018**
	Other government	1.03	0.495	2.16	0.931
	Private insurance	0.756	0.571	1	0.501
	Not insured	1.01	0.61	1.66	0.98
Facility type	Academic/research program	1	–	–	–
	Community Cancer Program	1.16	0.885	1.53	0.278
	Comprehensive Community Cancer Program	0.949	0.802	1.12	0.541
	Integrated Network Cancer Program	0.914	0.746	1.12	0.384
Tumor location	Right	1	–	–	–
	Left	1.35	1.14	1.61	** < 0.001**
	Transverse colon	1.38	1.1	1.73	**0.005**
	Overlapping lesion	1.06	0.619	1.82	0.83
Tumor size		1	1	1	**0.047**
Clinical TNM stage	I	1	–	–	–
	II	1.25	1.06	1.47	**0.007**
	III	1.47	1.23	1.76	** < 0.001**
Grade	Well-differentiated	1	–	–	–
	Moderately differentiated	0.96	0.769	1.2	0.72
	Poorly differentiated	1.19	0.909	1.56	0.206
	Undifferentiated	1.93	1.28	2.9	**0.002**
Histology	Adenocarcinoma	1	–	–	–
	Mucinous adenocarcinoma	1.1	0.873	1.38	0.419
	signet-ring cell carcinoma	0.942	0.495	1.79	0.856
Type of colectomy	Segmental colectomy	1	–	–	–
	Subtotal /hemicolectomy	1.25	1.05	1.48	**0.011**
	Total colectomy	2.06	1.37	3.09	** < 0.001**
	Total proctocolectomy	0.842	0.291	2.44	0.751
	Colectomy, non-specified	0.97	0.512	1.84	0.926
Resection of contiguous organ	No	1			
	Yes	1.9	1.49	2.42	** < 0.001**
Approach	Laparoscopic	1	–	–	–
	Robotic-assisted	0.501	0.393	0.637	** < 0.001**

*CI* confidence interval

Bold text in *p* value column indicates statistical significance

Different clinical scenarios were applied to the predictive calculator and the risk of conversion was calculated. The worst-case scenario that included all predictors of increased conversion (male with stage III left-sided undifferentiated adenocarcinoma who underwent laparoscopic total colectomy with resection of contagious organs) had a predicted probability of conversion of 0.947, corresponding to a combined OR of 17.8. The combined odds of conversion in this scenario were reduced by half to 8.9 when the robotic-assisted platform was used (Supplementary Figure).

### Impact of conversion on short-term outcomes and survival

Conversion to open surgery was associated with higher 30-day (2.8% vs 1.4%, *p* < 0.001) and 90-day mortality (4.8% vs 2.3%, *p* < 0.001), 30-day unplanned readmission (5.5% vs 4%, *p* < 0.001), a longer median hospital stay (5 vs 4 days, *p* < 0.001), and a higher incidence of positive surgical margins (5.5% vs 2.4%, *p* < 0.001) (Table 4).

**Table 4:** Impact of conversion on outcomes

Factor	Group	No conversion (*n* = 23,721)	Conversion (*n* = 2825)	*p*-value
30-day mortality (%)	Yes	273 (1.4)	68 (2.8)	** < 0.001**
	No	19,338 (98.6)	2337 (97.2)	
90-day mortaliy (%)	Yes	452 (2.3)	114 (4.8)	** < 0.001**
	No	19,030 (97.7)	2277 (95.2)	
30-day readmission (%)	No readmission	22,272 (94.6)	2611 (92.7)	** < 0.001**
	Planned readmission	282 (1.2)	38 (1.3)	
	Unplanned readmission	931 (4.0)	154 (5.5)	
	Planned and unplanned readmission	46 (0.2)	13 (0.5)	
Median hospital stay in days (IQR)		4.00 [3.00, 5.00]	5.00 [3.00, 7.00]	** < 0.001**
Surgical margins (%)	Positive	572 (2.4)	154 (5.5)	** < 0.001**
	Negative	23,055 (97.6)	2662 (94.5)	
Overall survival (%)	Alive	16,431 (83.0)	1832 (75.6)	** < 0.001**
	Dead	3363 (17.0)	592 (24.4)	

*IQR* interquartile range

Bold text in p value column indicates statistical significance

**Fig. 2:**
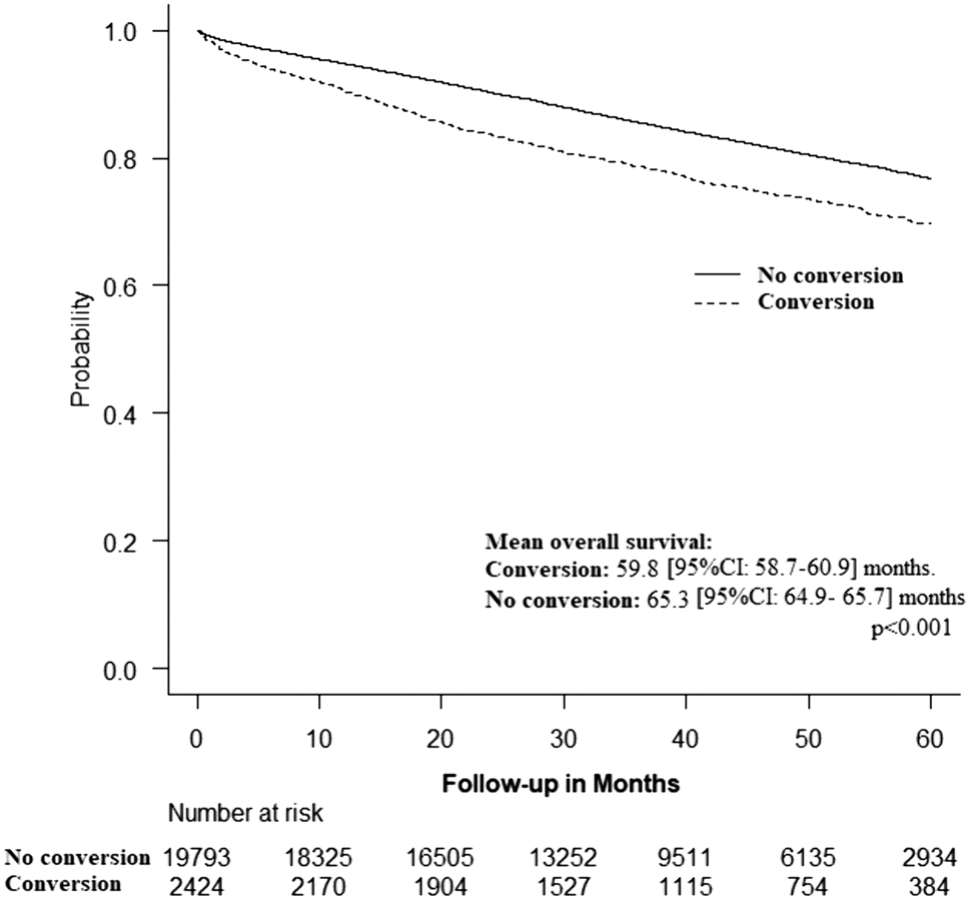
Kaplan–Meier curve illustrating the difference in overall survival between converted and non-converted patients

Kaplan–Meier analysis revealed that conversion was associated with shorter mean OS duration (59.8 [95%CI 58.7–60.9] months vs 65.3 [95%CI 64.9–65.7] months, *p* < 0.001) (Fig. 2).

## Discussion

The present study found that 10.6% of minimally invasive colectomies were converted to open surgery; this rate was similar to the rate reported by Tekkis et al. [15], yet lower than the conversion rates (15–18%) reported in previous literature. This difference is perhaps because the previous studies analyzed conversion in the setting of colon and rectal surgery together, whereas our study assessed conversion only in minimally invasive colectomy. Furthermore, right-sided colectomy, known to be associated with lower conversion rates [7], accounted for half of the cases in the present study. Predictors of conversion included patient and tumor-related factors such as sex, tumor side, size, grade, and stage and treatment-related factors as the extent of resection and surgical approach. Each of these factors may be independently associated with an increased risk of conversion to laparotomy, and thus should be carefully considered within the operative plan.

Male sex was associated with a 19% increase in the odds of conversion to open colectomy. A previous analysis of the University Health System Consortium administrative database [6] also demonstrated a higher likelihood of conversion in men with an odds ratio of 1.2. Another study found male sex to be significantly associated with conversion from laparoscopic to open colorectal surgery [7]. While the increased incidence of conversion in male patients undergoing proctectomy is expected given the technical challenges associated with the narrow android pelvis [16], the increased odds of conversion of minimally invasive colectomy in men are entirely clear. This finding might be attributable to the body habitus and visceral adiposity in men that may render colectomy more challenging and increase the likelihood of conversion [17].

As was previously documented, resection of left-sided colon cancers was associated with 35% higher odds of conversion compared to resection of right-sided cancers. Other investigators [7] have reported a conversion rate of 21.4% for left colectomy versus 12.9% for right colectomy. Similarly, Simorov et al. [6] reported that transverse and left-sided colectomies had higher rates of conversion (20.8%) than that of right colectomy (15.6%). This finding is reasonable given the more complex anatomy of the transverse and left colon. The need for splenic flexure mobilization, which can be technically demanding, in left colectomy is consistent with the higher rates of complications and longer hospital stay compared to right colectomy, as previously noted [18].

A larger and more advanced colon cancer can result in a greater need for conversion from minimally invasive to open colectomy. Compared to stage I disease, stage II and III disease may increase the odds of conversion by 25% and 47%, respectively. This observation was in agreement with Biondi and colleagues [19] who found advanced-stage colorectal cancer associated with more than seven times the risk of conversion than early-stage tumors. It is plausible that bulky and advanced colon cancers may be more challenging to handle using a minimally invasive approach. However, evidence shows that MIS is safe and feasible in advanced colorectal cancers, including T4 tumors, and thus patients with locally advanced colon cancers should still be considered eligible for MIS [20].

Consistent with the previous findings, extended colectomy and the need for resection of a contiguous organ, which may indicate a more advanced disease, were associated with a higher risk of conversion to open surgery. Resection of a contiguous organ infiltrated by the primary colon cancer would increase the odds of conversion by 90%. Additionally, performing a total colectomy, for either a synchronous cancer or an associated condition such as diverticular disease, might double the risk of conversion. Although extended resections are typically associated with more conversions to open surgery, it would still be advisable to attempt MIS in these patients because even if they had to be converted to open surgery, they will have better outcomes than patients who had planned open surgery [7]. It may be recommended to use the robotic platform for patients at increased risk of conversion since it may reduce the risk of conversion by 50% as compared to laparoscopy. The lower rates of conversion with robotic-assisted surgery have been previously reported [8, 9, 21] and may be explained by the stable robotic platforms including 3-D vision and articulating instruments [22].

It is imperative to preoperatively and early intra-operatively anticipate conversions as they may have an adverse impact on short- and long-term outcomes. Conversion was associated with increased short-term mortality and unplanned readmission and may extend hospital stay by one day on average. These findings reinforce the conclusions of a previous study [16] about higher complications,in-hospital mortality, and longer stay when patients were converted to open surgery. We assume that conversion has a negative impact on oncologic outcomes as it was associated with shorter OS. This finding was concordant with another study [19] that concluded that conversion results in worse cumulative DFS and OS than does successful laparoscopic completion.

Based on the findings of this study, patients in whom the risk of conversion to open surgery is expected to be higher than average, such as male patients, patients with bulky and advanced tumors, and patients undergoing extended resections and en block resections, might benefit from the robotic platform in reducing the odds of conversion.

The main strength of the present study is using data from a large national database. National databases can provide a reliable source of big data that can help reduce sampling bias. The multi-center source of the data also improves the external validity and generalizability of the findings, in contrast to single-center studies where the findings may not necessarily apply to other hospitals or regions. Another point of strength and novelty of our study is the use of ChatGPT to write an R code for developing a predictive calculator for conversion. The development of predictive statistical calculators using the R language can be a long and demanding process, however ChatGPT provided the required code within minutes. Although it needs to be externally validated, the developed calculator may be used to calculate the risk of conversion to open surgery and may help tailor the surgical approach to individual patients according to the anticipated risk of conversion before surgery.

The main limitations of the present study include the retrospective nature of the data and, thus, the risk of selection bias. The data used in the analysis were derived from CoC-accredited hospitals only, and thus non-CoC accredited hospitals are not represented and the validity of the predictors for conversion needs to be examined in other settings. Data on the clinical stage of colon cancer were missing for several patients, which led to the exclusion of many potentially eligible patients. The lack of a standard definition of the main outcome of the study, conversion to open surgery, is another limitation since it is possible that different hospitals used varying definitions of conversion. In addition, other factors that may impact the risk of conversion such as body mass index, intra-operative complications, blood loss, hospital volume, and threshold and definition of conversion were not accounted for in the model used in this study. Another limitation is the inability to evaluate surgeons’ technical skills. Finally, the results of the present study are pertinent only to colon cancer as the study did not include other diagnoses. The findings of the present study need to be interpreted with caution considering the many above-mentioned limitations, namely the lack of data on other possible predictors for conversion. Thus, sensitivity of the developed calculator may not be optimal, yet it can be improved by adding other possible predictors in future iterations.

## Conclusions

Male patients and those with bulky, high-grade, advanced-stage, and left-sided colon cancers are at increased risk of conversion from minimally invasive to open colectomy. While patients undergoing extended resections and en block resection of a contiguous organ have a greater likelihood of conversion, the use of the robotic platform may be associated with a reduced likelihood of conversion. The integration of the above-mentioned risk factors into a predictive risk calculator may help reduce the incidence of conversion to open surgery, however external validation of our predictive calculator is needed.

### Supplementary Information

Below is the link to the electronic supplementary material.

Supplementary Figure: Risk calculator to predict conversion from minimally invasive to open surgery

Supplementary file2 (DOCX 14 KB)

## Data Availability

Data used in the study will be available by the corresponding author upon reasonable request.
