# Supplementary figures and images for: An artificial intelligence-designed predictive calculator of conversion from minimally invasive to open colectomy in colon cancer
Source: Updates Surg. 2024 Jun 26;76(4):1321–30. doi: 10.1007/s13304-024-01915-2 (PMC11341585; doi:10.1007/s13304-024-01915-2)

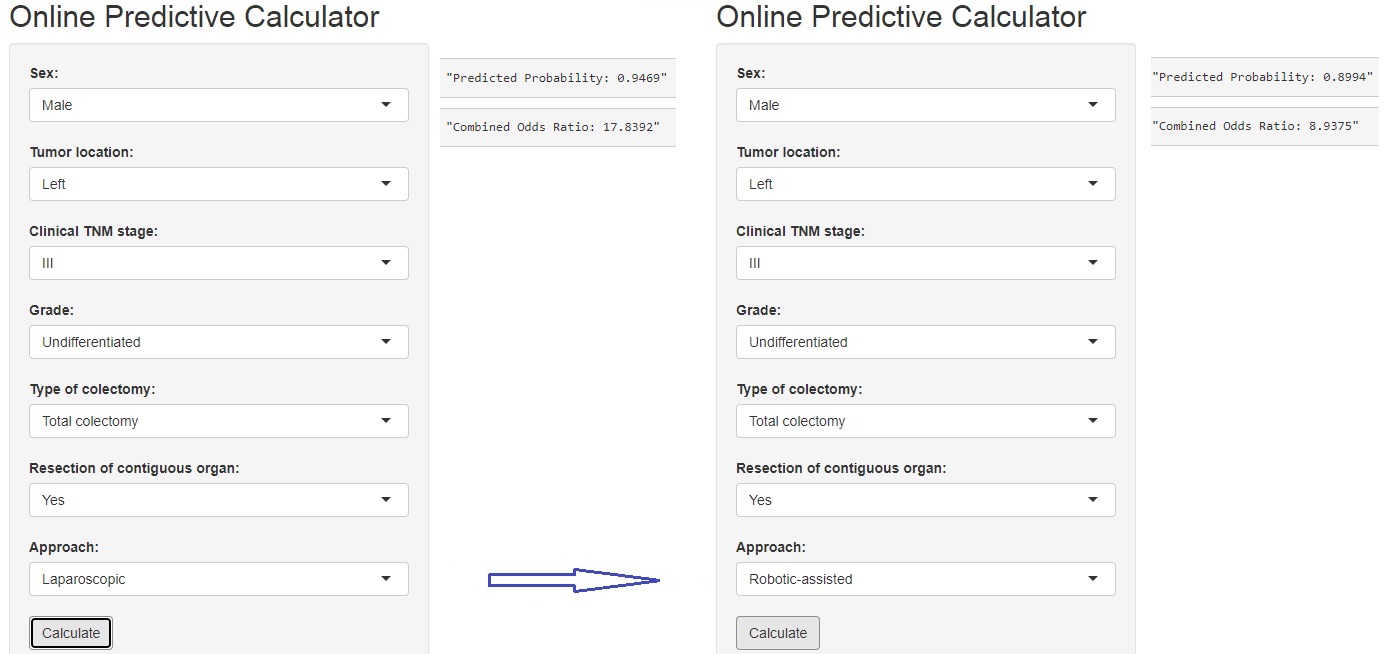

Supplement: Supplementary file 1 — Supplementary Figure: Risk calculator to predict conversion from minimally invasive to open surgery [file 13304_2024_1915_MOESM1_ESM.jpg]
