# Supplementary material for: An artificial intelligence-designed predictive calculator of conversion from minimally invasive to open colectomy in colon cancer
Source: Updates Surg. 2024 Jun 26;76(4):1321–30. doi: 10.1007/s13304-024-01915-2 (PMC11341585; doi:10.1007/s13304-024-01915-2)
Supplement: Supplementary file 2 — Supplementary file2 (DOCX 14 KB) [file 13304_2024_1915_MOESM2_ESM.docx]

library(shiny)

# Define UI

ui <- fluidPage(

titlePanel("Online Predictive Calculator"),

sidebarLayout(

sidebarPanel(

selectInput("sex", "Sex:", choices = c("Female", "Male")),

selectInput("tumor", "Tumor location:", choices = c("Right", "Left", "Transverse colon", "Overlapping lesion")),

selectInput("stage", "Clinical TNM stage:", choices = c("I", "II", "III")),

selectInput("grade", "Grade:", choices = c("Well-differentiated", "Moderately differentiated", "Poorly differentiated", "Undifferentiated")),

selectInput("colectomy", "Type of colectomy:", choices = c("Segmental colectomy", "Subtotal /hemicolectomy", "Total colectomy", "Total proctocolectomy", "Colectomy, non-specified")),

selectInput("resection", "Resection of contiguous organ:", choices = c("No", "Yes")),

selectInput("approach", "Approach:", choices = c("Laparoscopic", "Robotic-assisted")),

actionButton("calculate", "Calculate")

),

mainPanel(

verbatimTextOutput("result"),

verbatimTextOutput("odds_ratio")

)

)

)

# Define server

server <- function(input, output) {

# Reactive expression to calculate the combined odds ratio and predicted probability

calculateResults <- reactive({

# Define the odds ratios

odds_ratios <- list(

sex = c(Female = 1, Male = 1.19),

tumor = c(Right = 1, Left = 1.35, `Transverse colon` = 1.38, `Overlapping lesion` = 1.06),

stage = c(I = 1, II = 1.25, III = 1.47),

grade = c(`Well-differentiated` = 1, `Moderately differentiated` = 0.96, `Poorly differentiated` = 1.19, Undifferentiated = 1.93),

colectomy = c(`Segmental colectomy` = 1, `Subtotal /hemicolectomy` = 1.25, `Total colectomy` = 2.06, `Total proctocolectomy` = 0.842, `Colectomy, non-specified` = 0.97),

resection = c(No = 1, Yes = 1.9),

approach = c(Laparoscopic = 1, `Robotic-assisted` = 0.501)

)

# Calculate the log-odds

log_odds <- log(odds_ratios$sex[[input$sex]]) +

log(odds_ratios$tumor[[input$tumor]]) +

log(odds_ratios$stage[[input$stage]]) +

log(odds_ratios$grade[[input$grade]]) +

log(odds_ratios$colectomy[[input$colectomy]]) +

log(odds_ratios$resection[[input$resection]]) +

log(odds_ratios$approach[[input$approach]])

# Calculate the predicted probability

probability <- exp(log_odds) / (1 + exp(log_odds))

# Calculate the combined odds ratio

combined_odds_ratio <- exp(log_odds)

list(probability = probability, combined_odds_ratio = combined_odds_ratio)

})

# Display the predicted probability

output$result <- renderPrint({

if (input$calculate > 0) {

result <- calculateResults()

paste("Predicted Probability:", round(result$probability, 4))

}

})

# Display the combined odds ratio

output$odds_ratio <- renderPrint({

if (input$calculate > 0) {

result <- calculateResults()

paste("Combined Odds Ratio:", round(result$combined_odds_ratio, 4))

}

})

}

# Run the Shiny app

shinyApp(ui, server)
